# Effects of Minute-Scale Preaging Time on Formability of AA2195-T34 Alloy

**DOI:** 10.3390/ma19061089

**Published:** 2026-03-12

**Authors:** Yanling Dai, Nanhui Peng, Jianguo Gong, Chongrui Xu, Lihua Zhan, Wenxia Yang

**Affiliations:** 1 College of Aeronautical Manufacturing, Jiangxi Aviation Vocational and Technical College, Fuzhou 344000, China; 2 School of Chemical Engineering, Jiangxi Normal University, Nanchang 330022, China; 3 Light Alloy Research Institute, Central South University, Changsha 410083, China; 4 State Key Laboratory of High-Performance Complex Manufacturing, Central South University, Changsha 410083, China; 5 College of Life Sciences, Gannan Normal University, Ganzhou 341000, China

**Keywords:** 2195 Al-Li alloy, formability, creep-age forming, minute-scale preaging, microstructures

## Abstract

**Highlights:**

- Minute-scale preaging at 200 °C shows an optimum at 5 min, minimizing yield ratio (65.55%) and maximizing elongation (17.86%).
- MSP ≤ 8 min increases ultimate creep strain (maximum 0.23% at 5 min) and delays peak aging without reducing peak strength.
- Microstructurally, MSP5 reduces GP-zone density, retards T_1_ precipitation, and enhances dislocation mobility; MSP15 promotes T_1_ and lowers ductility.
- MSP provides a practical pre-treatment to improve CAF formability of AA2195-T34 while retaining peak-aged mechanical performance.

**Abstract:**

The influence of minute-scale preaging (MSP) at 200 °C on the microstructures and formability of 2195-T34 Al-Li alloy in the creep-aging (CA) process was systematically investigated. The elongation of the alloy before CA first increased and then decreased with MSP time (2 to 15 min), reaching a maximum of 17.86% at 5 min; the yield ratio exhibited the opposite trend, attaining a minimum of 65.55% at 5 min. When the true strain was greater than 1.4%, the strain hardening rate of the samples after MSP treatment for less than 8 min was similar to and higher than that of the samples preaged for 15 min. Compared with the non-preaged specimens, the ultimate creep deformation of samples preaged for less than 8 min was improved, reaching a maximum improvement of 29.5% at 5 min. Simultaneously, the peak aging was retarded without reducing the final peak strength (T6 level), thereby broadening the formability window. HAADF-STEM observations revealed that MSP for 5 min markedly reduces the density of G.P. zones, which delays T_1_ precipitation and reduces the resistance to dislocation motion. When the preaging time was less than 8 min, the number of movable dislocations increased. However, exceeding 8 min led to obvious precipitation of the T_1_ phase, reducing the plasticity of AA2195-T34 sheets.

## 1. Introduction

With the rapid development of the aerospace industry, more and more attention is paid to the requirement for reducing the weight of components and parts. The development of lightweight and high-strength materials is an effective method that can be used to achieve this requirement. In comparison with conventional aluminum alloys, Al-Li alloys exhibit a lower density together with a higher elastic modulus and superior specific stiffness [1,2,3]. However, Al-Li alloys are very difficult to precisely form owing to their poor plasticity and pronounced elastic recovery at room temperature [4]. Creep-age forming (CAF) has been developed to manufacture large-scale components, particularly those featuring complex geometries and high structural stiffness [5,6]. CAF is a process that adds an external stress onto the material based on an artificial aging process, which can give full play to the formability of alloys and effectively improve their mechanical properties [7]. Previous studies have revealed the dependency of creep formability and age hardening of Al-Li alloys on aging parameters. The creep strain level increases significantly with increasing creep-aging temperature. A higher creep-aging temperature accelerates the evolution of dislocations toward an equilibrium state [8]. The application of external stress accelerates precipitation while leading to a more homogeneous distribution of precipitates in creep-aged alloys [9]. Jiang et al. [10] found that the threshold stress determines the mechanism by which external stress promotes the precipitation of precipitated phases. This promotion can be attributed to lattice distortion induced by elastic stress, together with the enhanced dislocation multiplication resulting from plastic deformation. Fu et al. [11] studied the role of the thermal–mechanical loading sequence on the creep formability of 5A90 Al-Li alloy. The creep deformation and toughness for the loading–heating sequence are larger than those for the heating–loading sequence. Zhan et al. [12] found that an increase in the loading rate leads to an increase in creep deformation due to dislocation evolution.

**Figure 1:**
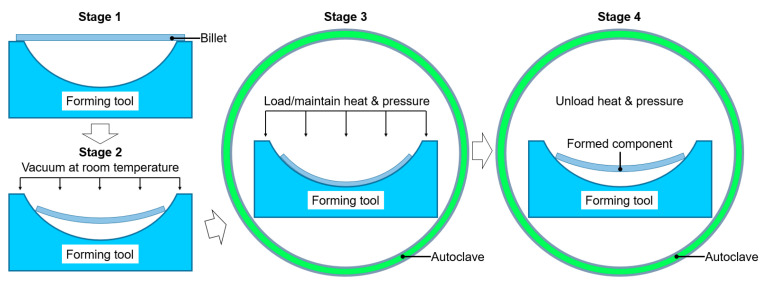
Typical creep-age forming process using autoclave technology.

To enhance and accelerate the creep-aging response, the influence of pretreatment prior to aging on creep aging has been investigated. Dong et al. [13] studied the relationship between the microstructure evolution and mechanical properties of AA2195 alloy predeformed by rolling with different reductions. Increasing the predeformation can introduce a large number of dislocations into the lattice, which can detach from the pinning effect of strengthening precipitates. Wang et al. [14] found that predeformation shortens the time of primary creep and raises the steady-state creep rate. The precipitates are promoted and more dispersed owing to the predeformation, which enhances the mechanical properties of the Al–Li–S4 alloy. However, it cannot be ignored that directly applying atmospheric pressure to the billet by external vacuum pumping is one of the important steps in the actual operation processes of CAF using an autoclave. A certain level of plastic deformation occurs, and cracks may initiate in the billets of thin-walled components with complex structures at room temperature. The actual operation process of CAF is shown in Figure 1 [15]. First, the original billet is placed on the forming mold. Second, the vacuum bag and the forming tool surface form a closed cavity, in which the alloy billet is located, and the cavity becomes a vacuum at room temperature. Third, pressure and heat are applied to make the billet contact with the mold surface. Finally, rebound occurs after unloading, and a panel component is obtained. For the production of large-sized parts, the heating/loading time in the autoclave can account for 40% of the whole process time at most. Age hardening also takes place in this process, which reduces the forming capacity of the plate. Nevertheless, the existing literature ignores the requirement for room-temperature loading and long-term loading formability at Stage 2 and Stage 3 of CAF. In recent years, preaging has been explored as an effective approach to regulate creep-aging behavior. Zeng et al. [16] reported that preaging at 120 °C for several hours improved the post-creep strength of Al–Zn–Mg–Cu alloys, but did not address its influence on formability. Fangpu et al. [17] proposed a combined process of “preaging (165 °C for 6–24 h) and pre-straining” for Al–Cu alloys, which enhanced precipitation strengthening and creep strain; however, the prolonged thermal exposure extended the production cycle. Chen et al. [18] investigated the effects of preaging at 65–115 °C for various durations on Al–Li alloys, and found that higher temperatures reduced subsequent creep strain, yet the formability window was not evaluated. Overall, existing approaches are either excessively time-consuming and incompatible with industrial energy-efficiency requirements or fail to address the critical degradation of formability. Therefore, developing a short-time pretreatment method that is highly compatible with the CAF process, enhances formability in Stages 2 and 3, and preserves final strength is of significant importance.

This study proposes a minute-scale preaging treatment, which enhances formability without compromising mechanical properties or extending the CAF cycle, thereby offering both energy-saving and high-efficiency advantages. Based on this approach, the effects of minute-scale preaging treatment on the formability of AA2195 alloy during the actual whole CA process and MSP treatments with different process parameters were investigated. The microscale and nanoscale microstructures, such as grain, textures, precipitates and dislocations before CA and after CA, are characterized and analyzed. The relationship between the microstructure and formability in the creep-aging process was revealed. The influence of MSP treatment on stress aging hardening behavior was analyzed.

## 2. Experimental Methods and Materials

**Figure 2:**
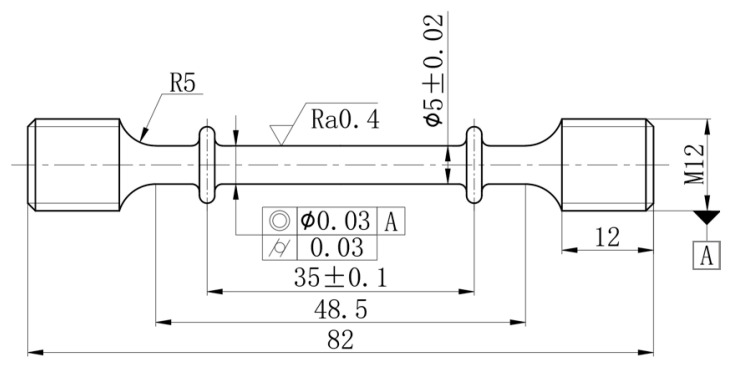
Dimensions of the samples.

The specimens shown in Figure 2 were machined from an AA2195 alloy rolled plate along the rolling direction, with a thickness of 8 mm, and provided by the Beijing Institute of Astronautical Systems Engineering (Beijing, China). The chemical composition of the test alloy was measured using a plasma spectrometer (SPECTRO Analytical Instruments GmbH, Kleve, Germany), and the results are listed in Table 1. Minute-scale preaging was conducted in an air-circulating furnace preheated to 200 °C. After the furnace temperature stabilized, the specimens were placed inside and held for 2, 5, 8, or 15 min, followed by water quenching. These conditions are denoted as MSP2, MSP5, MSP8, and MSP15, respectively. The room-temperature formability of the alloy under different preaging conditions was evaluated by measuring the strain hardening rate (SHR), yield ratio, and elongation of the uniaxial tensile deformation [19,20]. Then, the uniaxial tension creep strain was tested at 180 °C for 18 h under 220 MPa (lower than the yield strength of 280 MPa at 180 °C) in the RMT-D10 high temperature creep test machine (Zhuhai Sansi Taijie Electrical Equipment Co., Ltd., Zhuhai, China). The room temperature (25 °C) mechanical properties before and after creep aging were obtained by tensile testing on an MTS-5105 machine with an accuracy of ±1 °C under the rate of stretching of 2 mm/min. Each test was repeated three times.

The grain structure was tested by electron backscattered diffraction (EBSD) in an OXFORD instrument (Oxford Instruments, Abingdon, UK) at an acceleration voltage of 20 kV. EBSD data were analyzed using HKL Channel 5 software with a scan step size of 0.5 μm. To investigate the precipitation behavior, high-angle annular dark-field scanning transmission electron microscopy (HAADF-STEM) observations were conducted with a Titan G2 60-300 (FEI Company, Eindhoven, The Netherlands) operated at 300 kV. STEM and EBSD specimens were prepared by mechanically polishing samples to a thickness of 80–100 μm, and then they were thinned using a twin-jet technique with 30% HNO_3_ and 70% CH_3_OH solution at approximately −45–−30 °C [21]. X-ray diffraction (XRD) analysis was performed using an Advance D8 X-ray diffractometer (Bruker AXS SE, Karlsruhe, Germany) at a speed of 2°/min [22]. Three representative areas were scanned per condition, yielding a total characterized area of 3 × 150 × 150 μm^2^ per condition.

## 3. Results and Discussion

### 3.1. Formability

**Figure 3:**
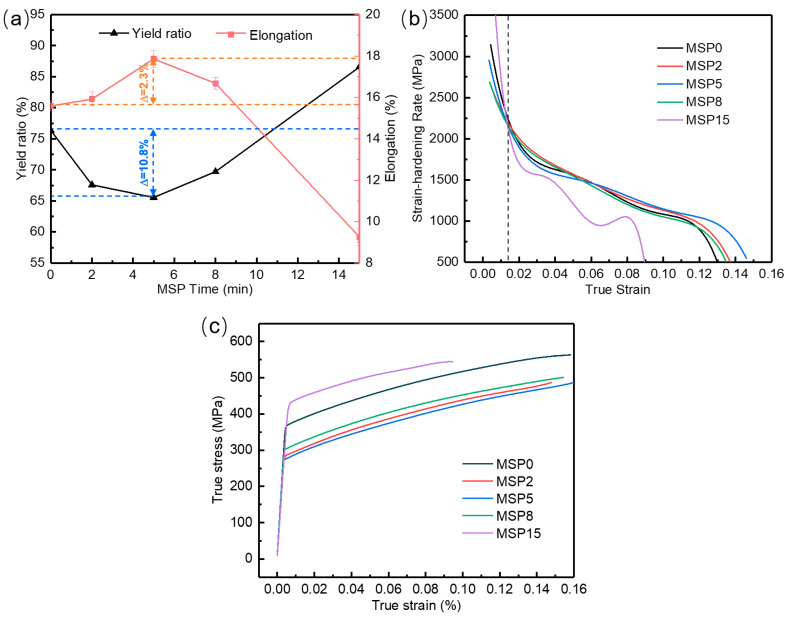
Yield ratio and elongation (**a**), strain hardening rate (**b**), and true stress–true strain curves (**c**) with various MSP times at 200 °C.

The yield ratio, elongation, and strain hardening rate of the specimens after MSP treatment are compared in Figure 3. Generally, the lower the yield ratio is, the better the plasticity [23]. This parameter is therefore considered an important indicator for evaluating the formability of aluminum alloys. The results demonstrate that the yield ratio first decreases and then increases with the MSP time, exhibiting a pronounced nonlinear variation overall. The minimum value is 65.55% when the preaging time is 5 min. The elongation first increases and then decreases, reaching a maximum value (17.86%) at 5 min, suggesting that the MSP5 sample obtains its highest tensile ductility and strain hardenability. The strain hardening rate gradually decreases with increasing strain due to the reduction in the ability of the alloy sample to store dislocations, a trend that is consistently observed under all treatment conditions. In addition, when the true strain value exceeds 1.4%, the strain hardening rates of the samples after MSP treatment for less than 8 min are similar and remarkably greater than that of the MSP15 sample, which indicates that the AA2195-T34 alloy after preaging treatment for 15 min at 200 °C exhibits a low ductility [24]. As shown in Figure 3a–c, the minute-scale preaging time has a pronounced effect on the strength–ductility balance of the material. Among the conditions investigated, the MSP5 treatment achieves a more favorable compromise between yield ratio and elongation.

**Figure 4:**
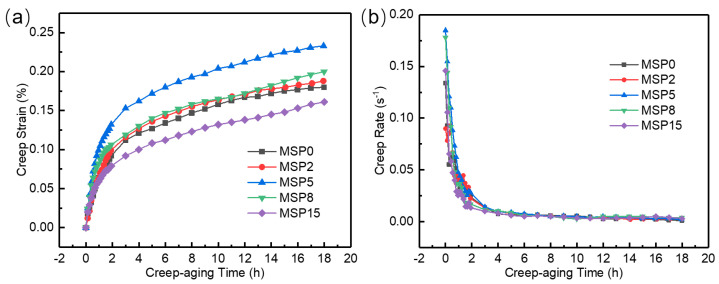
Creep strain (**a**) and creep strain rates (**b**) with various MSP times under an external tensile stress of 220 MPa.

Figure 4 shows the creep-aging curves and creep strain rate curves of the AA2195 MSP specimens at 180 °C under 220 MPa for 18 h with various short-term preaging times. These curves exhibit typical two-stage creep characteristics, which are divided into the creep first stage and the creep second stage by the change in creep rate [25]. Throughout the creep process, strain continues to accumulate with time, while the differences among the specimens are mainly manifested at the initial stage. These differences indicate the role of the preaging treatment in tailoring the microstructural state of the material. With a higher MSP time, the ultimate creep strain increases first and then decreases, and the largest value is 0.23% at 5 min, caused by different creep rates at the initial creep stage. The steady creep strain after MSP treatment for 2, 5, and 8 min increased by 4.5%, 29.5%, and 11.1%, respectively, compared with the as-received alloy. Nevertheless, after MSP treatment for 15 min, the steady creep strain decreased by 10.6%. These trends suggest that minute-scale preaging has a marked influence on the deformation behavior of the alloy during the creep-aging process. Accordingly, appropriate control of the preaging time can be an effective approach to achieving a more desirable creep response and improved forming performance.

**Figure 5:**
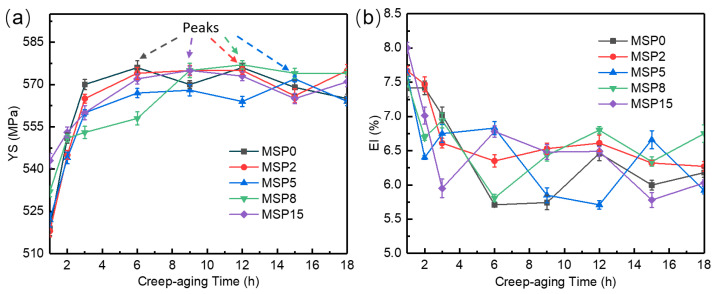
YS (**a**) and El (**b**) of the AA2195 MSP specimens after CA at 180 °C under an applied stress of 220 MPa.

Figure 5 exhibits the yield strength (YS) and elongation (El) curves of the AA2195 MSP specimens after CA at 180 °C under an applied stress of 220 MPa. The aging hardening behavior of specimens under five different minute-scale preaging conditions exhibited a similar trend: the YS increased rapidly in the creep early stage, then increased slowly, and finally stabilized. In contrast, the elongation first decreased and subsequently tended to stabilize with increasing aging time. After creep-aging at 180 °C, the MSP0 sample reached its peak strength at 6 h with a YS of 576 MPa, consistent with the typical T6 temper of AA2195 reported in the literature [26]. Of note, we observed a prolongation in the creep time required to reach the maximal strength of the samples after minute-scale preaging treatments, but the mechanical properties of peak-aged samples were all essentially the same. This was different from the effect of long-term preaging treatment. Prolonged preaging led to a modest enhancement in strength, while simultaneously promoting a faster precipitation process during the subsequent aging stage [27]. Since large-scale components are formed by CAF, a long heating and boosting stage cannot be ignored, and aging hardening also occurs [28]. Peak aging generally corresponds to a low elongation; thus, delaying peak aging is conducive to enlarging the high formability window in the creep-aging process. Taken together, short-term preaging for less than 8 min at 200 °C can improve formability in the creep aging process.

### 3.2. Grain Feature

**Figure 6:**
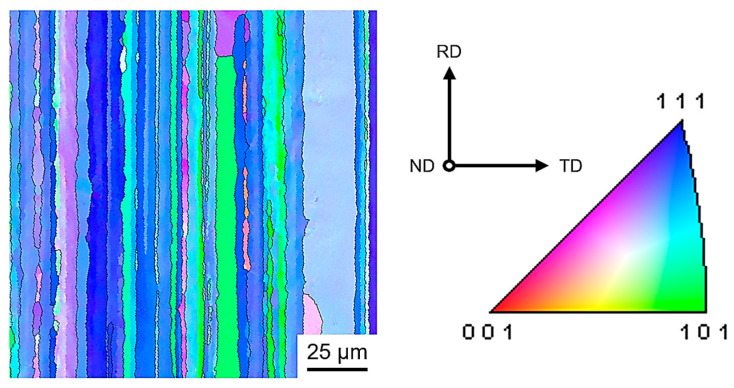
IPF maps of the MSP5 specimen after CA for 18 h under 220 MPa at 180 °C.

**Figure 7:**
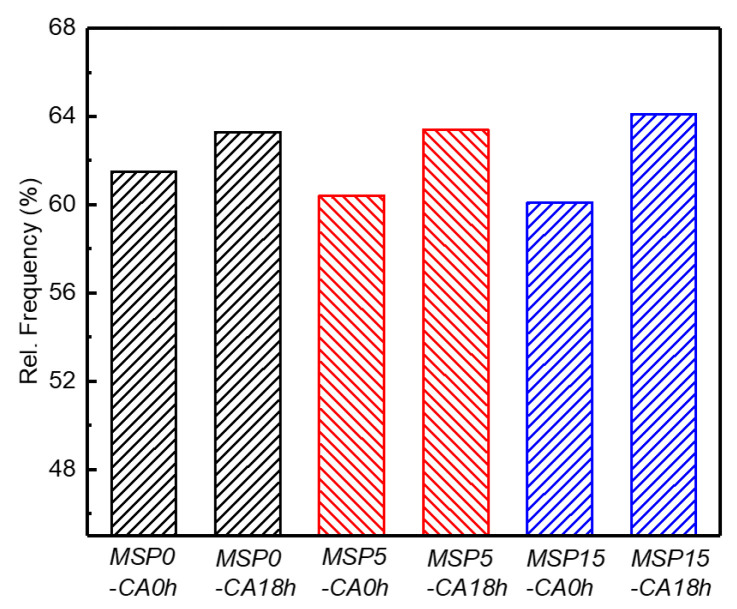
Frequency distribution of the LAGBs under different conditions.

Referencing the inverse pole figure (IPF) coloring scheme, different colors represent different grain orientations in EBSD maps. The spatial variation in crystallographic orientation can therefore be directly assessed from the color distribution. There are a lot of elongated-shaped grain structures along the RD direction in the samples, showing a pronounced directional morphology. Most of these grains exhibit a (111) crystallographic orientation. This shows that the samples have typical microscopic characteristics of rolled aluminum alloys [29]. As shown in Figure 6, the difference in the grain size is significant, and the grain morphology exhibits clear inhomogeneity across different regions. It is also observed that many small grains exist, resulting from the fracture of rolled fibers due to large compression deformation. These fine grains are predominantly distributed within the original grains or in the vicinity of the grain boundaries. High-angle grain boundaries (HAGBs) and low-angle grain boundaries (LAGBs) (2° < θ < 15°) are commonly used to categorize grain boundaries and subgrain boundaries [30]. The LAGB distribution of the 2195 Al-Li alloy treated by different methods is shown in Figure 7. The LAGBs of the 2195-T34 alloy decrease slightly with the MSP time, while the difference in the LAGB of the MSP samples after CA for 18 h decreases. Increasing creep deformation results in a higher frequency of low-angle grain boundaries.

**Figure 8:**
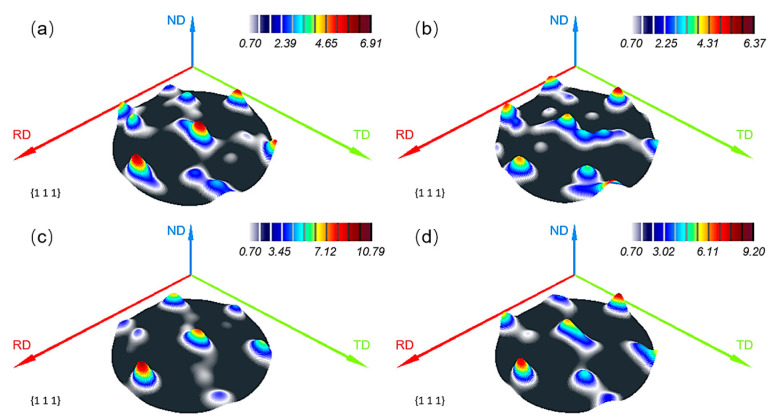
{111} 3D pole figures of (**a**) MSP0 and (**b**) MSP5 samples after CA for 0 h and (**c**) MSP0 and (**d**) MSP5 samples after CA for 18 h.

During plastic deformation, polycrystalline metals tend to develop a preferred crystallographic orientation among the constituent grains [31]. Such orientation evolution is generally closely associated with the direction of the applied load and the specific mode of deformation. Figure 8 shows the {111} 3D pole figures from the 2195-T34 aluminum alloy under different conditions. The pole figures provide an intuitive representation of the grain orientation distribution and its corresponding evolution behavior. The alloy has a typical rolling texture, the densities of which hardly change with preaging, indicating that short preaging treatments have a limited influence on the initial texture.

The texture densities of the age-formed samples gradually increase with the extension of creep-aging time, suggesting that the creep process plays a dominant role in driving texture evolution. A similar phenomenon has been found in 7XXX Al alloys [32]. Because the grains with abundant orientation are unstable, the initial stress applied during CA will make the grains rotate towards a stable direction, thereby altering the overall orientation distribution. As a result, with increasing creep deformation, the texture density increases with increasing aging time, contributing to aging hardening, which has a significant influence on the macroscopic mechanical properties of the material.

### 3.3. Dislocation Characteristics

**Figure 9:**
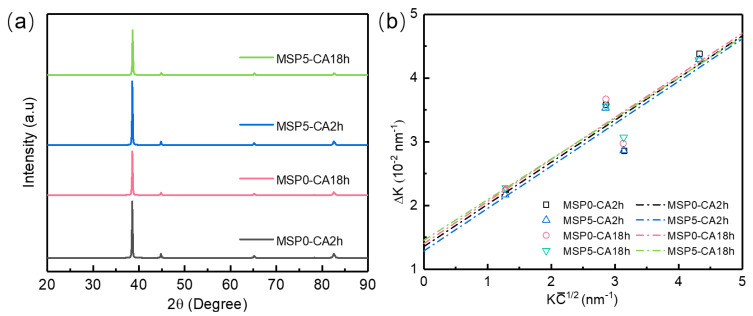
(**a**) XRD patterns and (**b**) the modified Williamson-Hall plot of the samples.

**Figure 10:**
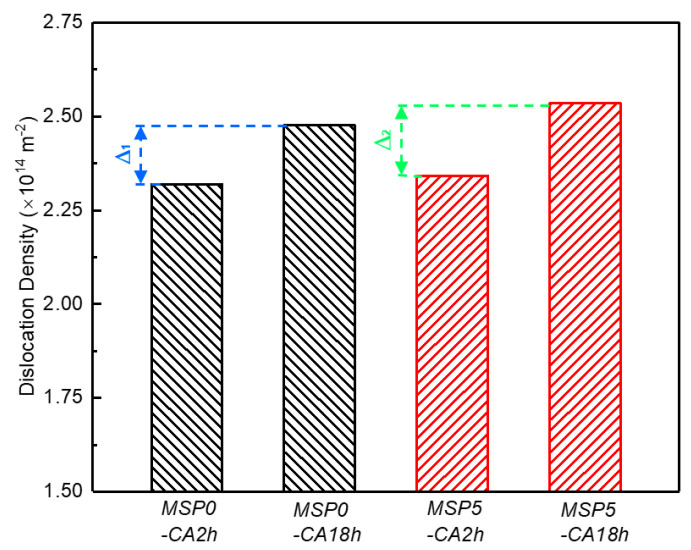
Dislocation density of the samples under different conditions.

The multiplication and annihilation of dislocations, related to the number of dislocation motions and their interactions, directly affect the dislocation density. In order to quantitatively investigate the dislocation density evolution during creep deformation, the XRD test [33] was performed on the specimens. Based on the modified Williamson-Hall method [34], the dislocation density of the AA2195 alloy under different conditions was calculated. Figure 9b shows the best linear fitting between Δ*K* and $K{\bar{C}}^{1/2}$. C represents the average contrast factor [35]. ΔK refers to the broadened full width at half-maximum (FWHM) [36]:

(1) $$\left\{\begin{array}{c} \Delta K\;=\;0.9/d+\left(\frac{\pi A^{2}b^{2}}{2}\right)\rho_{t}^{\frac{1}{2}}K{\bar{C}}^{1/2}\\ \Delta K\;=\;\frac{(2\Delta \theta )cos\;\theta_{i}}{\lambda }\\ K\;=\;2sin\;\theta /\lambda \\ \bar{C}\;={\bar{\;C}}_{h00}\left\{1-qH^{2}\right\} \end{array}\right.$$

where *θ* and 2Δ*θ* refer to the diffraction angle and FWHM of the diffraction peak at *θ*, respectively, obtained from Figure 9a. *λ* is the X-ray wavelength, *λ* = 0.154 nm. *A* and *b* = 0.286 nm represent the material constant and burgers vector [37]. Figure 10 presents the dislocation density of the samples. Under initial tensile stress, the dislocation density increases with creep aging. This is because the coherent strain between the growing precipitates and the matrix will become more and more significant as the creep aging progresses [38]. Some studies have shown that the number of mobile dislocations is often positively correlated with the dislocation density. When there are more mobile dislocations, the creep deformation is higher. Furthermore, the MSP treatment has little influence on the dislocation density of the MSP specimens during the CA process. Therefore, we speculate that the difference in the creep strain may be due to the number of movable dislocations affected by the interaction between dislocations and precipitates.

### 3.4. Precipitate Behavior

**Figure 11:**
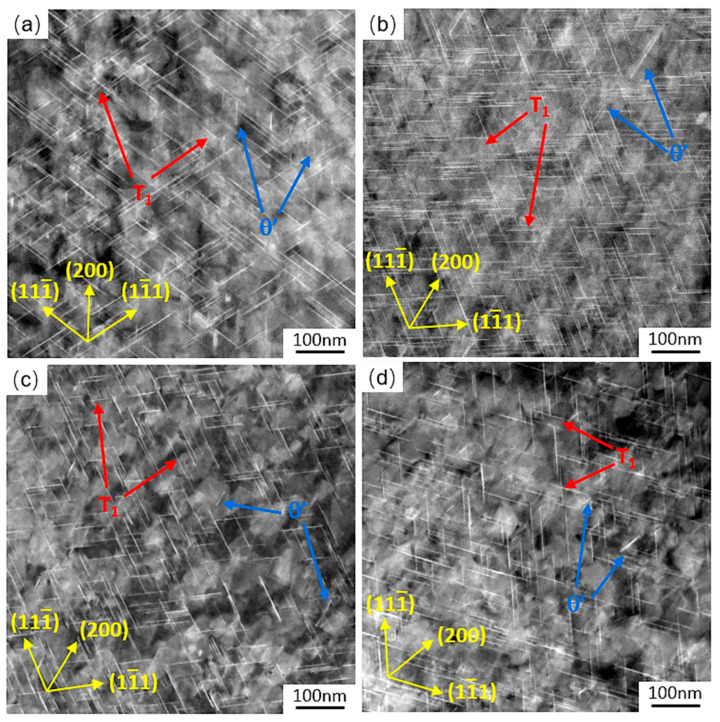
HAADF-STEM images of the samples treated with (**a**) MSP0, (**b**) MSP5 after CA for 2 h under the initial stresses of 220 MPa at 180 °C, and (**c**) MSP0 and (**d**) MSP5 after CA for 18 h. All images were taken from the <011> matrix direction.

The HAADF-STEM micrographs of precipitates in AA2195 alloy samples after CA are illustrated in Figure 11. This microscopic characterization technique allows the morphology and spatial distribution of the precipitated phases to be clearly and directly observed. It can be seen that the microstructure of AA2195 alloy is composed of an α-Al matrix and disk-like T_1_ [39] and θ’ [40] precipitates, which exhibit distinct length-scale characteristics within the matrix. Due to the small density of θ’ precipitates, which were insufficient for promoting creep behavior change, this study mainly discusses the T_1_ phase. The distribution of T_1_ precipitates (marked by the red arrow) is uniform and dispersed, and their morphological features play a critical role in determining the alloy’s mechanical response.

**Figure 12:**
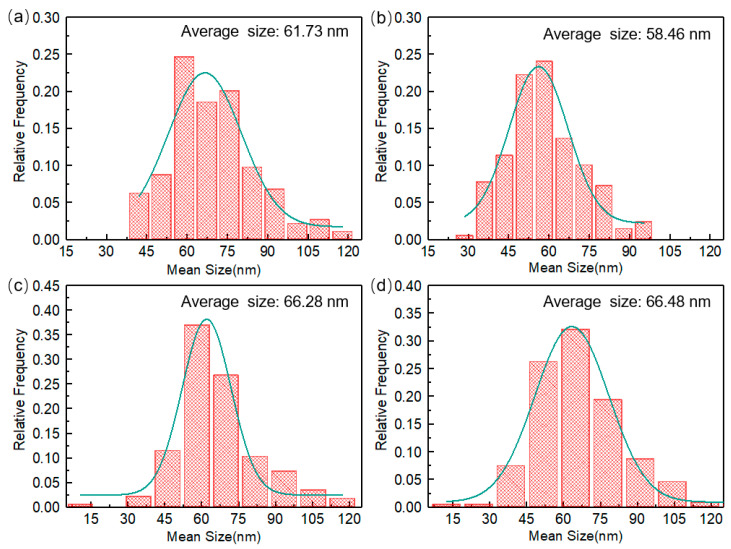
The size distribution of the T_1_ precipitate for (**a**) MSP0 and (**b**) MSP5 after CA for 2 h under an initial stress of 220 MPa at 180 °C, and (**c**) MSP0 and (**d**) MSP5 after CA for 18 h.

To quantitatively evaluate the effect of minute-scale preaging on T_1_ precipitates in prestrained Al-Cu-Li alloys treated with MSP0 and MSP5 after CA under the initial stresses of 220 MPa at 180 °C, the size distribution and mean sizes of T_1_ precipitates were measured using Image-Pro Plus. To guarantee statistical validity, at least three STEM diagrams were selected, and more than 200 precipitates were counted, thereby minimizing the influence of random error. The difference in the T_1_ phase mean sizes of the samples treated with MSP0 and MSP5 creep aging for 2 h is shown in Figure 12a,b. Figure 12 shows that when samples are short-term preaged for 5 min, the relative volume fraction of large precipitates in the alloys is less than that for alloys treated with MSP0 during the primary creep regime. In contrast, the volume fraction of small precipitates of MSP5 is larger than that of MSP0. Different minute-scale preaging times have a significant effect on the evolution of the precipitated phase during the CA process. It is interesting that as the stress aging time is extended to 18 h, the mean size of the T_1_ phase at steady-state creep aging of the alloy under different preaging conditions stabilizes at approximately 66 nm, and the size distribution exhibits little difference, as illustrated in Figure 12c,d. Minute-scale preaging has no obvious effect on the size, distribution, and quantity of the precipitated phase at the steady creep stage, consistent with the law of mechanical properties of the alloy in the steady-state creep stage. Therefore, the minute-scale preaging treatment delays the precipitation of precipitates but will not affect the final precipitation size and distribution.

**Figure 13:**
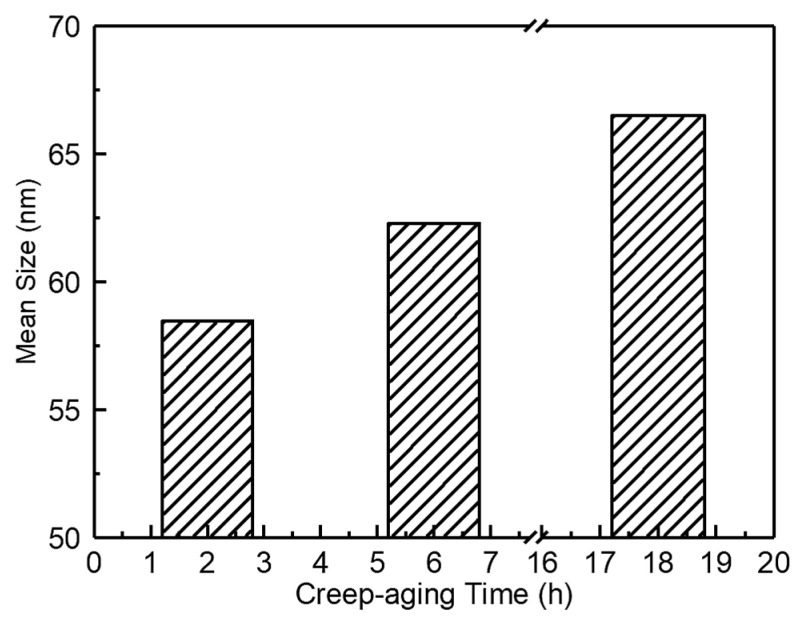
The average size of the T_1_ precipitate for MSP5 specimens after CA for different times under 220 MPa at 180 °C.

Figure 13 shows the change in the T_1_ phase mean sizes of the MSP5 sample with increasing creep-aging time. As shown in Figure 13, when the creep time increases from 2 h to 6 h, the average size of the T_1_ phase increases by 6.53%. When the creep time increased to 18 h, the average size of the T_1_ phase increased by 6.74%. As the aging duration further increased, the T_1_ phase in the alloy coarsened to some extent. The closer to the steady-state creep stage the alloy is, the longer it takes for the average size of the precipitated phase to grow to the same extent.

### 3.5. The Influence of MSP Treatment on Microstructure and Formability

The initial applied stress promotes the dislocation to overcome the obstacle in the early creep stage, causing the creep deformation to increase rapidly during the CA process. As the aging time progresses, the precipitation strengthening increases and the growth of precipitates effectively hinders the movement of dislocations, so the rapid reduction in creep strain rate occurs. Since the dynamic dislocation density is directly determined by the competition between the dislocation increment and dislocation recovery, when the multiplication and recovery of dislocations reach a dynamic equilibrium, the dislocation density reaches the maximum, and the evolution rate tends to zero. It is strange that minute-scale preaging will slightly reduce the LAGB in the MSP samples. Since most low-angle grain boundaries are composed of dislocations and dislocation walls [32], the decrease in the LAGBs is due to the recovery of some dislocations during the preaging process. Based on a model described by Taylor [41], the annihilation rate of dislocations in MSP samples is low at room temperature, and the dislocation storage rate dominates the hardening rate. The minute-scale preaging makes the dislocations annihilate, resulting in decreasing in dislocation slip resistance. With the increase in dislocation recovery rate, the strain hardening rate of the material in the MSP15 sample decreases, although there is a certain amount of precipitate in the MSP15 sample. However, compared with the dislocation increment during rapid creep aging, the dislocation recovery of the MSP specimen has little effect on the creep strain, as shown in Figure 10.

**Figure 14:**
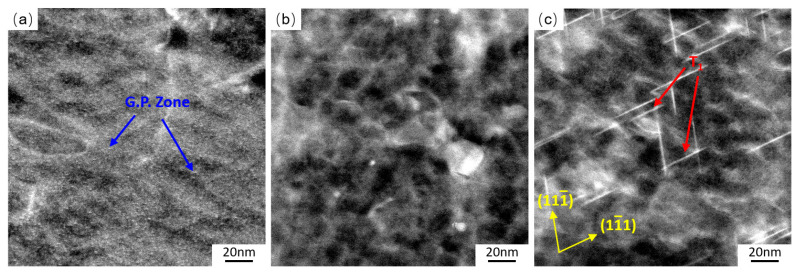
HAADF-STEM images of 2195-T34 Al-Li alloy treated with (**a**) MSP0, (**b**) MSP5, and (**c**) MSP15. All images were taken from the <011> matrix direction.

Cu atom is one of the components of different precipitated phases of AA2195 alloy. The amount of Cu atoms existing in the free state is definite, which inevitably leads to the precipitation competition during aging hardening [42]. Section 3.4 confirmed that T_1_ becomes the main precipitated phase, which has the greatest strengthening effect and can effectively inhibit coplanar slip [43]. The precipitate sizes of the samples treated with different minute-scale preaging times are apparently different during the CA process. Short-term preaging for less than 8 min delays the precipitation of precipitates, leading to a significant prolongation of the time required to achieve peak strength. Through further HADDF-STEM observation (Figure 14), it was found that the solute aggregates (G.P. zone) exhibit a much brighter contrast distribution uniformly in the 2195-T34 Al-Li samples (MSP0). The Cu-rich clusters are the monatomic layer of copper, similar to the GPI region usually forming in Al-Cu alloys [44]. Their density correlates with the dislocations caused by the prestrain. The strain field induced by G.P. zones in the matrix interacts with dislocations, affecting the movement and configuration of dislocations and easily causing uneven local deformation, deteriorating the forming ability [45]. The G.P. zone in the initial state of the alloy dissolves after minute-scale preaging for 5 min, as shown in Figure 14b. Therefore, under the same dislocation density of the MSP specimens, the dissolution of G.P. zones can increase the number of activated slip systems. The more movable dislocations there are, the better the formability is in the whole CAF process. Notably, when the MSP treatment time exceeds 8 min, the T_1_ phase in the sample precipitates, which will weaken the formability.

## 4. Conclusions

The current study was designed to investigate the effects of the minute-scale preaging time on the formability of AA2195-T34 alloy in the CAF process. A systematic analysis was carried out by correlating mechanical properties, creep behavior, and microstructural evolution, with the aim of providing both theoretical guidance and experimental support for the optimization of processing parameters. The following conclusions were obtained:

(1) Formability improvement governed by an optimal MSP duration. An appropriate MSP treatment markedly enhances CAF formability, particularly when the MSP duration does not exceed 8 min at 200 °C. This improvement is evidenced by variations in the yield ratio, elongation, and achievable creep strain. The yield ratio of the AA2195-T34 alloy first decreases and then increases with the MSP time, and the elongation exhibits the opposite trend. This behavior suggests the presence of an optimal processing window that promotes more effective plastic accommodation. Correspondingly, the ultimate creep deformation of the MSP samples after creep aging at 180 °C under 220 MPa first increases and then decreases with MSP time, reaching a maximum at 5 min, which was 29.5% higher than that of the non-preaged sample.
(2) The creep-aging of AA2195-T34 alloy after MSP treatment at 180 °C exhibits two-stage behavior, consisting of an initial rapid deformation stage followed by a relatively steady deformation stage. The minute-scale preaging treatment slowed the progression of aging hardening, while the peak-aged strength remains largely unaffected. As a result, the deformation capability is improved without sacrificing the overall strength level. The delayed peak aging broadened the high formability window throughout the CAF process, which is beneficial for the accurate forming of complex components.
(3) The enhanced formability originates from MSP-induced modifications in microstructural evolution, particularly in terms of clustering behavior, precipitation kinetics, and dislocation activity. The MSP for less than or equal to 8 min dissolves the G.P. zones in the AA2195-T34 alloy, and the T_1_ phase during the CA process is delayed, which weakens the movement resistance of dislocations and increases the number of movable dislocations, which is the fundamental mechanism responsible for the improvement in forming performance.

## Figures and Tables

**Table 1:** The chemical composition of the studied AA2195-T34 alloy (in wt. %).

Cu	Li	Mg	Ag	Zr	Fe	Ti	Si	Mn
3.95	1.09	0.34	0.26	0.13	0.037	0.036	0.019	0.0016

## Data Availability

The original contributions presented in this study are included in the article. Further inquiries can be directed to the corresponding author.
